# Diffusion tractography of post-mortem human brains: Optimization and comparison of spin echo and steady-state free precession techniques

**DOI:** 10.1016/j.neuroimage.2011.09.054

**Published:** 2012-02-01

**Authors:** Karla L. Miller, Jennifer A. McNab, Saad Jbabdi, Gwenaëlle Douaud

**Affiliations:** a FMRIB Centre, Nuffield Department of Clinical Neurosciences, University of Oxford, Oxford, UK; b A.A. Martinos Centre, Massachusetts General Hospital, Boston, USA

**Keywords:** Diffusion, Tractography, Post mortem, Steady-state free precession, DTI

## Abstract

Diffusion imaging of *post-mortem* brains could provide valuable data for validation of diffusion tractography of white matter pathways. Long scans (*e.g.*, overnight) may also enable high-resolution diffusion images for visualization of fine structures. However, alterations to *post-mortem* tissue (T2 and diffusion coefficient) present significant challenges to diffusion imaging with conventional diffusion-weighted spin echo (DW-SE) acquisitions, particularly for imaging human brains on clinical scanners. Diffusion-weighted steady-state free precession (DW-SSFP) has been proposed as an alternative acquisition technique to ameliorate this tradeoff in large-bore clinical scanners. In this study, both DWSE and DW-SSFP are optimized for use in fixed white matter on a clinical 3-Tesla scanner. Signal calculations predict superior performance from DW-SSFP across a broad range of protocols and conditions. DW-SE and DW-SSFP data in a whole, *post-mortem* human brain are compared for 6- and 12-hour scan durations. Tractography is performed in major projection, commissural and association tracts (corticospinal tract, corpus callosum, superior longitudinal fasciculus and cingulum bundle). The results demonstrate superior tract-tracing from DW-SSFP data, with 6-hour DW-SSFP data performing as well as or better than 12-hour DW-SE scans. These results suggest that DW-SSFP may be a preferred method for diffusion imaging of *post-mortem* human brains. The ability to estimate multiple fibers in imaging voxels is also demonstrated, again with greater success in DW-SSFP data.

## Highlights

► Comparison of DW-SE and DW-SSFP for post-mortem imaging on clinical scanners. ► Optimization of protocols predicts 50-130% higher SNR efficiency in DW-SSFP. ► Comparison of tractography 6- and 12-hour DW-SE and DW-SSFP scans. ► Lower uncertainty on fibre direction in DW-SSFP produces superior tractography. ► Crossing fibres can be estimated from 12-hour DW-SSFP data.

## Introduction

Diffusion-weighted imaging (DWI) is a commonly used magnetic resonance imaging (MRI) technique for brain imaging that capitalizes on the sensitivity of water diffusion to a number of tissue properties of interest. One such property is the dependence of diffusion-weighted signal on the orientation of white-matter fibers, which enables white matter tracts to be reconstructed using diffusion “tractography” techniques (Basser et al., 2000; Conturo et al., 1999; Mori et al., 1999). The relatively indirect link between water diffusion and the pathways of interest makes validation of these methods particularly important. Nevertheless, surprisingly little validation has been reported to date. One possibility would be to perform DWI scans of *post-mortem* tissue, which could be compared to various invasive methods for fiber determination, including classical dissection, histological fiber visualization or (in animals) tracer techniques (Dyrby et al., 2007; Leergaard et al., 2010; Schmahmann et al., 2007). The ability to scan samples for long periods of time could further enable high spatial resolution for reconstructing small tracts or fine tract features (D'Arceuil et al., 2007, 2008; McNab et al., 2009; Miller et al., 2011).

One key challenge to MRI of *post-mortem* brains is the alterations in tissue properties due to death and fixation. For diffusion imaging, the most important alterations are reduced apparent diffusion coefficient (ADC) (Sun et al., 2003) and T_2_ (Pfefferbaum et al., 2004; Tovi and Ericsson, 1992). Despite these changes to the tissue diffusion properties, tractography in *post-mortem* brains has been demonstrated by several groups (D'Arceuil et al., 2007; Dyrby et al., 2007; McNab et al., 2009). The primary issue with changes to ADC is therefore the need for higher b-values to obtain equivalent contrast to *in-vivo* studies, while changes in T_2_ directly impact the achievable signal level.

Conventional diffusion imaging methods on clinical scanners using diffusion-weighted spin-echo (DW-SE) are poorly-suited to imaging of fixed tissue. In DW-SE of *post-mortem* tissue, the need for higher b-value generally requires longer echo times, which results in very low signal levels due to the short T_2_. A number of studies have previously acquired diffusion-weighted data in whole, *post-mortem* human brains (Larsson et al., 2004; Pfefferbaum et al., 2004; Schmierer et al., 2007) or brain slices (Gouw et al., 2008). However, these studies have only made modest increases in b-value (b = 750–2000 s/mm^2^) and used large voxel size to increase SNR. As a result, the image quality and diffusion contrast in these studies are often worse than that achievable *in-vivo*. Straightforward application of protocols developed for *in-vivo* imaging has ultimately proved insufficient to obtain the quality of data required for the applications discussed above.

Several groups have performed *post-mortem* diffusion imaging using small-bore pre-clinical scanners, studying *post-mortem* animal brains (D'Arceuil et al., 2007, 2008; Dyrby et al., 2007; Guilfoyle et al., 2003; Kroenke et al., 2005; Tyszka and Frank, 2009; Verma et al., 2005), spinal cord (Kim et al., 2009; Schwartz et al., 2005) and human tissue sections (D'Arceuil et al., 2005; Guilfoyle et al., 2003). These scanners are typically equipped with high-performance gradients that are well-suited to diffusion imaging by enabling high b-value without requiring long T_E_. Unfortunately, whole, *post-mortem* human brains are too large to fit into these scanners, making clinical scanners the most likely alternative.

In this manuscript, we compare two techniques for diffusion imaging of whole, *post-mortem* human brains using a clinical 3 T MRI scanner. Specifically, we compare tractography results obtained from DW-SE and diffusion-weighted steady-state free precession (DW-SSFP), an alternative method recently proposed for *post-mortem* diffusion imaging by our group (McNab et al., 2009). We begin by contrasting the predicted SNR efficiency for these two methods in *post-mortem* tissue, concluding that the DW-SSFP sequence is expected to out-perform DW-SE due to its high SNR efficiency in tissues with short T_2_. We then present a comparison of diffusion data acquired on the same *post-mortem* human brain using optimized protocols compatible with an overnight scan (6- and 12-hour protocols). The DW-SSFP data exhibits lower uncertainty on the fiber orientation, which should directly lead to improved diffusion tractography. We present diffusion tractography in four major white matter tracts: the corpus callosum, corticospinal tract, cingulum bundle and superior longitudinal fasciculus. For all of the tracts considered, the DW-SSFP tracts were superior when scan times are matched, covering a larger spatial extent and in good agreement with known anatomy. Comparison of 6-hour DW-SSFP and 12-hour DW-SE data suggests that DW-SSFP performs at least as well as DW-SE if not better, despite being acquired in half the scan time.

## Diffusion imaging of fixed tissue

The goal of this section is to evaluate the expected performance of DW-SE and DW-SSFP sequences in *post-mortem* white matter. We begin by highlighting important changes to the MR properties of fixed tissue, discuss the potential approaches to diffusion imaging that are suited to these conditions, and finally present predicted SNR efficiency measures for the two sequences under consideration.

### MR properties of fixed tissue

Changes in the diffusion properties of fixed tissue are an important consideration for *post-mortem* imaging, particularly in light of their dependence on properties like the *post-mortem* interval and fixation protocol.

The ADC has been reported to be 2–5 times lower than *in-vivo* when tissue is fixed immediately after death (D'Arceuil et al., 2007; Sun et al., 2003, 2009), and even lower when fixation is delayed, as occurs for most human cadaver specimens (D'Arceuil and de Crespigny, 2007; Miller et al., 2011; Shepherd et al., 2009a). A factor of two reduction in ADC is expected for tissue scanned at room temperature (18–22 °C) rather than body temperature (37 °C) (D'Arceuil et al., 2007; Le Bihan, 1995; Thelwall et al., 2006). The further reductions observed in *post-mortem* experiments may be due to microstructural alterations due to tissue degradation and/or protein cross-linking with fixation. Although early reports found fractional anisotropy (FA) to be preserved in animal tissue (D'Arceuil et al., 2007; Guilfoyle et al., 2003; Sun et al., 2003, 2005), there is also evidence that it may be reduced with long *post-mortem* intervals (D'Arceuil and de Crespigny, 2007; Miller et al., 2011; Schmierer et al., 2007; Shepherd et al., 2009a).

Fixed tissue also has altered T_1_ and T_2_ compared to *in-vivo*, which appears to depend on specifics of the tissue preparation. Brain tissue stored (although not necessarily imaged) in fixative exhibits T_2_ = 35–50 ms at 1.5–4.7 T (D'Arceuil et al., 2007; McNab et al., 2009; Pfefferbaum et al., 2004; Yong-Hing et al., 2005). Soaking or rinsing fixed tissue in phosphate buffer (non-fixative) solution significantly increases T_2_ (D'Arceuil et al., 2007; Shepherd et al., 2009b; Thelwall et al., 2006), potentially even exceeding *in-vivo* values depending on the *post-mortem* interval (Shepherd et al., 2009a). These results suggest that short T_2_ is driven, at least in part, by the presence of fixative in tissue. Most studies have found reduced T_1_ of 350–500 ms reported in fixed white matter at field strengths of 1.5–4.7 T (D'Arceuil et al., 2007; McNab et al., 2009; Pfefferbaum et al., 2004; Yong-Hing et al., 2005), with a subtle dependence on *post-mortem* interval (Shepherd et al., 2009a). Washing with pure buffer solution appears to have little effect on T_1_ (D'Arceuil et al., 2007; Shepherd et al., 2009b), but gadolinium-doped buffer has been observed to reduce the T_1_ (D'Arceuil et al., 2007).

Soaking in buffer is thus an effective method to boost signal levels in animal brains and small tissue samples by increasing T_2_ and decreasing T_1_. Unfortunately, these approaches are more difficult in human brains due to their larger size, with both fixative (Yong-Hing et al., 2005) and buffer (D'Arceuil et al., 2007) taking at least a month to penetrate deep structures for human brains. Our preliminary investigations into buffer soaking resulted in a conspicuous boundary of buffer permeating only a few centimeters into human brains after 2–3 days (Miller et al., 2011). Soaking brains for extended periods risks degradation to exposed brain regions. We do not perform any buffer soaking, placing our experiments in the short-T_2_ and moderate-T_1_ regime.

For the rest of this manuscript, we will assume that fixed white matter at 3 T is characterized by T_2_ = 45 ms, T_1_ = 400 ms and D = 0.08 × 10^− 3^ mm^2^/s unless otherwise specified. These values are indicated by measurements in our tissue sample, but are also in good agreement with literature (D'Arceuil et al., 2007; McNab et al., 2009; Pfefferbaum et al., 2004; Yong-Hing et al., 2005).

### Diffusion imaging of post-mortem tissue

Although the changes to diffusion coefficient and T_2_ are generally problematic for diffusion imaging, other aspects of *post-mortem* imaging experiments are more favorable than *in-vivo*. The use of single-shot acquisitions for *in-vivo* diffusion imaging is dictated by motion sensitivity (Norris, 2001). The lack of motion in *post-mortem* scans enables the use of multi-shot acquisitions, which drastically reduces image artifacts from magnetic field inhomogeneities (due to both imperfect shim and the presence of air bubbles). Several previous studies have therefore proposed using the standard *in-vivo* DW-SE diffusion preparation with an altered readout module that acquires 3D k-space in multiple shots (D'Arceuil and de Crespigny, 2007; D'Arceuil et al., 2007; Miller et al., 2011; Tyszka and Frank, 2009). However, DW-SE preparations suffer from a trade-off between SNR (favoring short T_E_) and contrast (large b-value, requiring long T_E_), which become important when gradient strength is limited (*e.g.*, on clinical scanners). This trade-off is particularly problematic for the short T_2_ and low ADC of fixed tissue.

Recently, our group has proposed the use of diffusion-weighted steady-state free precession (DW-SSFP) (McNab et al., 2009), which is characterized by short T_R_ and the use of a single diffusion-encoding gradient (one lobe with duration *δ*). This sequence is difficult to use *in-vivo* due to its extreme motion sensitivity, but it may be ideal for *post-mortem* tissue. DW-SSFP is able to achieve diffusion weighting without requiring long T_E_ due to the cumulative effects of the diffusion gradient over multiple T_R_s, making it well-suited to the short T_2_ of fixed tissue. One complication of DW-SSFP is that the diffusion weighting depends on tissue T_1_ and T_2_, as well as flip angle (*α*) and T_R_. In addition to having a reasonable estimate of T_1_ and T_2_, this requires a different model for signal changes (Buxton, 1993; McNab and Miller, 2008) and makes the definition of a b-value inappropriate (since the signal attenuation depends on T_1_ and T_2_ as well as D). Nevertheless, to facilitate comparisons between DW-SE and DW-SSFP, we will refer to an “effective” b-value that reflects the diffusion contrast:

(1) $${\text{b}}_{\text{eff}}=-\frac{1}{D}ln\left(\frac{{\text{S}}_{\text{SSFP}}\left({\text{T}}_{\text{R}}\text{,}\;\alpha \text{,}\;\delta \text{,}\;D\text{,}\;{\text{T}}_{1}\text{,}\;{\text{T}}_{2}\right)}{{\text{S}}_{\text{SSFP}}\left({\text{T}}_{\text{R}}\text{,}\;\alpha \text{,}\;0\text{,}\;D\text{,}\;{\text{T}}_{1}\text{,}\;{\text{T}}_{2}\right)}\right)$$

where S_SSFP_ is the DW-SSFP signal for the given imaging (T_R_, *α* and *δ*) and tissue (T_1_, T_2_ and D) parameters. For the tissue of interest, the DW-SSFP sequence will have the same fractional signal attenuation as a DW-SE sequence with this b-value.

### Maximizing SNR efficiency

For the remainder of this section, we compare the performance of DW-SE and DW-SSFP in fixed tissue. We define optimal performance as the maximization of the signal-to-noise ratio (SNR) across all parameter settings that achieve a target b-value (or b_eff_). More specifically, we wish to optimize the *SNR efficiency*, which normalizes for the effect of scan duration and can therefore be used to predict the actual SNR for any given total scan time. Since SNR increases with the square root of scan time, SNR efficiency is given by:

(2) $$\eta =\frac{\text{SNR}}{\sqrt{{\text{T}}_{\text{scan}}}}\propto \frac{S\sqrt{N{\text{T}}_{\text{acq}}}}{\sqrt{N{\text{T}}_{\text{R}}}}=S\sqrt{\rho }$$

where *η* is the SNR efficiency, S is the raw signal level and the scan consists of *N* repetition periods (T_scan_ = *N*T_R_). During each T_R_, imaging data is collected over a readout duration T_acq_, such that $SNR\propto S\sqrt{N{\text{T}}_{\text{acq}}}$. We can rewrite this as a simple expression based on the “readout efficiency”, *ρ* = T_acq_ / T_R_, which is the fraction of each T_R_ that is dedicated to acquisition. This intuitive expression for the SNR efficiency, based on the raw signal level and the fraction of scan time spent actually acquiring data, provides a relatively simple optimization: maximize SNR efficiency subject to achieving a desired b-value^1^. (Note that the values calculated here are proportional to SNR efficiency and cannot be used to calculate actual SNR levels, since noise levels will in practice depend on hardware, such as receive coils and field strength.)

### Optimizing DW-SE

A few sequence parameters in DW-SE can reasonably be fixed in our optimization. Longer readouts (larger T_acq_) will generally increase SNR; however, in practice, readouts longer than 30 ms incur significant image distortion (and potentially T_2_* blurring artifacts, although we have not observed this artifact). We therefore fix the readout to this maximum acceptable duration: T_acq_ = 30 ms. At a given b-value, we also want to minimize the T_E_. Taking into account our T_acq_, the minimum achievable T_E_ on our scanner (with 5/8 partial Fourier factor) ranges from 91 to 148 ms for b = 1000–10,000 s/mm^2^. The key parameter that remains to be optimized is the T_R_ (assuming the optimal Ernst angle for the excitation pulse).

**Fig. 1:**
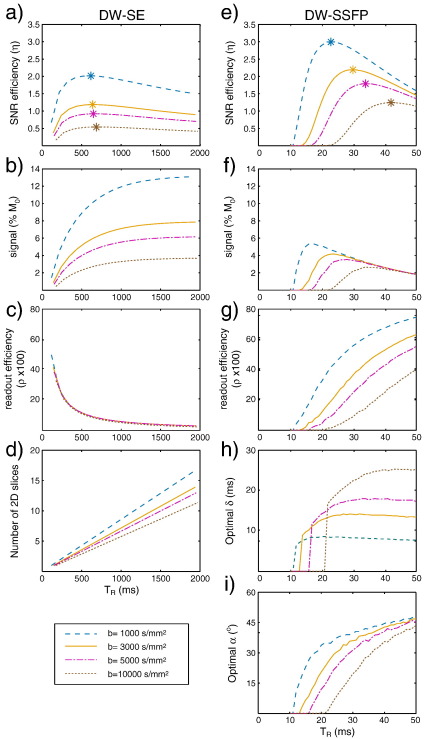
Sequence properties of DW-SE (a–d) and DW-SSFP (e–i) for b = 1000–10,000 s/mm^2^. We seek to maximize SNR efficiency. (a,e) Achievable SNR efficiency, calculated from the raw signal (b,f) and the readout efficiency (c,g). (b,f) Raw signal (in percent of M_0_), without diffusion weighting applied. DW-SE calculations use the minimum achievable T_E_ for each b-value on our scanner with a 5/8 partial Fourier factor. DW-SSFP calculations represent the optimal choice of diffusion gradient duration (*δ*, shown in h) and flip angle (*α*, shown in i) to achieve the desired b-value. (c,g) Readout efficiency (*ρ*) for each sequence, defined as the percent of each T_R_ during which data is acquired. For DW-SE, this calculation assumes a 30 ms EPI readout; DW-SSFP acquisitions are assumed to fill the remaining time in the T_R_ (and are always less than 30 ms). (d) In a 3D DW-SE sequence, any number of slices can be achieved, but for 2D sequences, the number of slices that can be achieved at the optimal T_R_ are severely limited.

The SNR efficiency calculations for DW-SE are presented in Figs. 1a–c assuming for our default tissue parameters (T_1_/T_2_ = 400/45 ms, D = 0.08 × 10^− 3^ mm^2^/s). It is particularly helpful to note how the signal and readout efficiency dependences on T_R_ interact to result in the final SNR efficiency: the optimal T_R_ reflects the best balance between high signal (favoring long T_R_ for T_1_ recovery) and efficient readout (for which T_acq_ must be a significant fraction of T_R_). The optimal T_R_ for DW-SE is roughly 500–700 ms (asterisks in Fig. 1a), considerably shorter than for *in-vivo* tissue (optimal range of 900–1100 ms) due to the shorter T_1_.

This range of T_R_ is reasonably compatible with 3D acquisitions, while 2D multi-slice acquisitions are unable to acquire very many slices in this window (as shown in Fig. 1d). In order to achieve whole brain coverage, a 2D multi-slice sequence would require considerably longer T_R_, drastically reducing the SNR efficiency. For example, our 3D protocols acquire 120 slices at b = 4500 s/mm^2^; a 2D DW-SE sequence would require T_R_ = 17 s to match slice coverage. Although the signal level is somewhat higher using the longer T_R_ (S_2D_ = 6.65 *vs* S_3D_ = 4.99) the difference in readout efficiency (*ρ*_2D_ = 0.18% *vs ρ*_3D_ = 3.92%) results in four times higher SNR efficiency in 3D (*η*_2D_ = 0.26 *vs η*_3D_ = 0.99). Each diffusion-weighted volume will take longer to acquire with segmented 3D readouts (*i.e.*, minutes rather than seconds), but this is readily compatible with the long scan times required to achieve usable SNR levels in *post-mortem* tissue. There is clearly considerable benefit in using 3D versions of DW-SE for *post-mortem* imaging.

### Optimizing DW-SSFP

For DW-SSFP, the optimization is slightly more complicated because the T_R_, duration of the diffusion gradient (*δ*) and flip angle (*α*) all affect the diffusion weighting. The optimization must therefore consider all three of these parameters. As with DW-SE, we impose a maximum readout duration T_acq_ ≤ 30 ms. However, in DW-SSFP the optimal T_R_ is quite short (20–40 ms), and high readout efficiency can often be achieved with a short T_acq_. Our optimizations therefore set T_acq_ based on the choice of T_R_ and *δ*, taking advantage of our ability to tailor the length of an EPI segment to match this optimized duration. We assume that the acquisition fills out all time during T_R_ that is not dedicated to diffusion weighting (*δ*) or other pulses (the “dead time”, T_dead_). In other words, we assume T_acq_ = T_R_ − *δ* – T_dead_ (using a conservative T_dead_ = 5 ms). Using these assumptions, we can optimize the SNR efficiency over every triplet of T_R_, *δ* and *α* that achieves a desired b_eff_.

This second set of optimizations is presented in Figs. 1e–i, based on the DW-SSFP signal equations derived by Buxton and colleagues (Buxton, 1993; Wu and Buxton, 1990). The shorter range of optimal T_R_ (20–40 ms) is due to the ability to obtain the target b_eff_ with a much shorter diffusion-weighting module. This leads to some crucial differences with DW-SE. First, the raw signal levels are much lower because the longitudinal magnetization has less time to recover (compare Figs. 1b and f, in which DW-SSFP has approximately half the signal of DW-SE). However, the ability to acquire diffusion-weighted signal at short T_R_ reduces the idle time in DW-SSFP, enabling much higher readout efficiency compared to DW-SE (which incurs long idle periods after acquisition waiting for the longitudinal magnetization to recover). For the parameters used in these calculations, the increased readout efficiency of DW-SSFP overcompensates for the reduced signal levels, and the SNR efficiency is predicted to be higher with DW-SSFP than DW-SE.

### Comparison of optimized DW-SE and DW-SSFP

Table 1 reports the peak SNR efficiency for the two sequences at a range of b-values assuming our default fixed tissue properties. DW-SSFP is predicted to exhibit 48–127% higher SNR efficiency than DW-SE, with greater gain at higher b-value. Naturally, the exact gains are dependent on the assumptions underlying our optimizations: most notably, the maximum acceptable T_acq_, the tissue properties (T_1_, T_2_ and D) and the achievable echo times in DW-SE. It is therefore useful to consider how the optimization is affected by these assumptions.

#### Readout duration

A longer T_acq_ would improve the readout efficiency of DW-SE, although at the expense of greater off-resonance artifacts (*e.g.*, distortion in EPI). If we increase the acceptable readout for DW-SE to T_acq_ = 40 ms, the expected gains range from 17 to 80% for b = 1000–10,000 s/mm^2^ (as above, greater gain at higher b-value). The DW-SSFP sequence at these b-values would still have short T_acq_ and therefore lower artifact levels (e.g., due to any air bubbles in the specimen).

#### Tissue properties

**Fig. 2:**
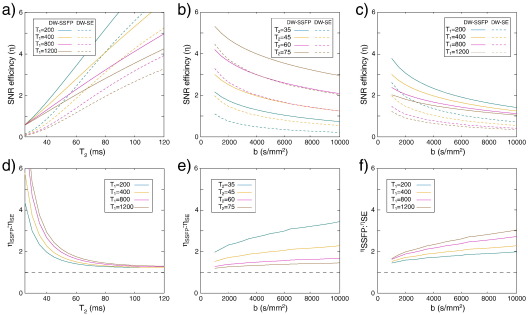
Dependence of the optimal SNR efficiency (*η*) for DW-SSFP and DW-SE on b, T_1_ and T_2_ values. The top row depicts the predicted SNR efficiency for DW-SE and DW-SSFP separately, while the bottom row shows the ratio of *η* for the two sequences (with the dashed line indicating equality and values greater than one indicating superior performance for DW-SSFP). For the tissue preparation presented here, values are expected to range from T_2_ = 35–50 ms and T_1_ = 350–500 ms. DW-SSFP is predicted to always out-perform DW-SE, with greatest benefit for long T_1_ and short T_2_, and at high b-value.

The optimizations in Table 1 assume our default T_1_ and T_2_ values, which differ from *in-vivo* tissue and depend on fixation protocols, as discussed above. In Fig. 2, we explore SNR efficiency across a broad range of T_1_, T_2_ and b values. In all cases, DW-SSFP is expected to out-perform DW-SE (as can be seen from the bottom row, where the ratio of *η* in DW-SSFP to DW-SE is always greater than one). Greatest gains are found for short T_2_, long T_1_ and high b-value. In light of the expected factor of 5–10 reduction in ADC for *post-mortem* tissue, experiments should generally be conducted at much higher b-value than *in-vivo*.

#### Diffusion preparation

The assumed echo times for DW-SE, based on the implemented sequence on our scanner, are lengthened by the twice-refocused preparation. Shorter TE could be achieved using the Stejskal–Tanner encoding scheme with a single refocusing pulse. Our signal calculations indicate that, in order to match SNR efficiency of DW-SSFP, we would need to reduce the echo times to approximately T_E_ = 74,88,96,112 ms for b = 1000,3000,5000,10,000 s/mm^2^, respectively. This may be achievable with a Stejskal–Tanner diffusion preparation and standard clinical hardware; however, it would come at the cost of higher eddy-current-induced distortions, which are already significant with our twice-refocused protocol (Miller et al., 2011). DW-SSFP data has been observed to have almost no eddy currents, presumably due to the reduced readout duration and short diffusion gradient.

#### Gradient heating

A final consideration relates to what the scanner can achieve in practice for DW-SSFP. The DW-SSFP sequence is highly gradient intensive, and the calculations above do not include any duty-cycle or heating calculations. For example, to achieve b_eff_ = 4500 s/mm^2^ (as in our experiments), the optimal DW-SSFP protocol would use T_R_/*δ* = 33/17.1 ms, which results in *η* = 1.87. Due to duty-cycle limitations, we found that we could achieve T_R_/*δ* = 42/16.7 ms, resulting in *η* = 1.68. Although this limits us in practice to 90% of the ideal DW-SSFP efficiency, it still represents a gain of 70% over the achievable SNR efficiency of DW-SE at this b-value (*η* = 0.99). We return to the duty-cycle limits imposed by gradient heating in the Discussion section.

## Methods

### Tissue preparation

Data was acquired on a whole, human brain with no known prior pathology (cause of death unknown). The brain was extracted from the cranium 24 h after death, and immersion fixed in a 10% neutral buffered formalin solution for 2 months prior to scanning. The brain was transferred to a close-fitting plastic container and immersed in a proton-free fluid (Fomblin LC08, Solvay Solexis Inc) that contributes no MR signal. Scanning was performed at room temperature (typically 19–21 °C). The brain was allowed to sit for 12 h prior to scanning to allow air bubbles to escape and the brain to reach thermal equilibrium. All scans were acquired in the same session to ensure good alignment and similar tissue deformation (which can be different if the brain is removed from the container between scan sessions).

### MRI scanning

#### Diffusion overview

**Fig. 3:**
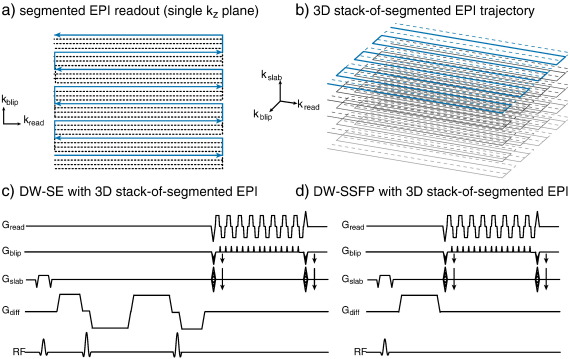
Diffusion-weighted spin echo and steady-state free precession sequences used in this study, both with a 3D segmented EPI acquisition module. (a, b) The trajectory consists of a segmented EPI trajectory in two dimensions (readout and phase-encode blip directions), which are stacked to fill out the third dimension of k-space (slab direction). (c) The DW-SE sequence uses this readout with a twice-refocused diffusion module. (d) The DW-SSFP sequence consists of an excitation, followed by a single diffusion-encoding gradient and then readout. Note that for simplicity of illustration, timings and gradient amplitudes are not to scale and elements such as EPI phase navigators and spoilers are not depicted.

All imaging was performed on a Siemens Trio 3 T scanner using a 12-channel head coil for signal reception. In the present work, we aimed to explore the potential for acquiring data in a single, overnight scan. For this reason, we report datasets acquired with shorter scans (6–12 h) than our 24-hour protocols reported previously (McNab et al., 2009; Miller et al., 2011). Both sequences use the system's maximum gradient amplitude (38 mT/m) to acquire 54 isotropically-distributed diffusion-encoding directions with b ≈ 4500 s/mm^2^. The diffusion-weighted sequences implemented in this work use 3D, segmented-EPI acquisitions, illustrated in Figs. 3a and b. This readout has high SNR efficiency due to 3D k-space coverage, and relatively short scan times for each image volume (a few minutes). In terms of distortion along the phase encode direction, the bandwidth (and echo spacing) is effectively scaled by the number of segments, as reported below. For typical field inhomogeneities (tens to hundreds of Hz maximum frequency offset), the diffusion scans are effectively distortion-less, enabling excellent co-registration with structural scans. Finally, the lack of motion in the tissue samples enables partial Fourier factors as low as 5/8 to reduce the T_E_ (which would incur significant artifacts *in-vivo* (Robson and Porter, 2005)).

#### DW-SE protocol

The diffusion-weighted spin echo (DW-SE) sequence was implemented with fairly minor modifications to the 2D single-shot EPI sequence used *in-vivo* (Fig. 3c). A twice-refocused diffusion-weighting scheme is used to reduce eddy current image distortions (Reese et al., 2003). The DW-SE scan was acquired with 0.94 × 0.94 × 0.94 mm^3^ resolution and diffusion weighting of b = 4500 s/mm^2^. Imaging parameters for this protocol include: T_E_/T_R_ = 122/530 ms, *α* = 75°, 789 Hz/pixel, 6 segments, matrix size 168 × 192 × 120, 5/8 partial Fourier factor, and acquisition time per volume 6 min and 22 s. The effective bandwidth (accounting for segmentation) is 4734 Hz/pixel (effective echo spacing 0.21 ms). A single repetition of this protocol (including six b = 0) scans takes 6.4 h; two repeats were acquired to enable a 12.8-hour data set to also be reconstructed. These datasets are referred to below as the “DW-SE-6” and “DW-SE-12” scans.

#### DW-SSFP protocol

Our implementation of DW-SSFP uses the same readout module as DW-SE with all gradient axes rewound to have zero net area (excluding the diffusion-weighting gradients). A single gradient lobe for diffusion weighting is inserted between the excitation and acquisition (Fig. 3d). The DW-SSFP protocol acquired 0.94 × 0.94 × 0.94 mm^3^ resolution, using T_E_/T_R_ = 28/42 ms, *α* = 37° and *δ* = 16.7 ms (assuming T_1_/T_2_ = 400/45 ms and D = 0.08 × 10^− 3^ mm^2^/s, this gives b_eff_ = 4470 s/mm^2^). Additional parameters include: 898 Hz/pixel, 11 segments, matrix size 180 × 192 × 120, 5/8 partial Fourier factor and acquisition time per volume 1 min and 15 s, effective bandwidth is 9878 Hz/pixel (effective echo spacing 0.10 ms). The diffusion-weighted protocol was repeated 10 times. In addition, 30 scans were acquired using T_E_/T_R_ = 13/27 ms, *α* = 37° and *δ* = 1.2 ms, leading to b_eff_ = 18 s/mm^2^. These images fulfill a similar function to b = 0 scans in a DW-SE sequence, although a small diffusion gradient is always necessary in DW-SSFP to act as a spoiler. Note that this low-b scan was acquired with shorter T_R_ to reduce total scan time and increase SNR; the difference in T_R_ is accounted for in the signal modeling. Analysis of DW-SSFP data included measured T_1_ and T_2_ values. These were acquired as described in previously (McNab et al., 2009), but using a subset of images that can be acquired in 32 min (rather than the 3-hour acquisition described previously). Specifically, we used two averages of SPGR at *α* = 7° and 37°, one IR-SPGR image with TI = 400 ms, and BSSFP images at *α* = 18 deg, 43° and 85° (with 0° and 180° phase cycling for each). The mean T_1_ and T_2_ values from a large white matter mask are input to the BEDPOST model fitting. As with the DW-SE data, we considered two DW-SSFP datasets: a 6.6-hour data set taken from 5 averages of the DW-SSFP data, and a 12.3-hour dataset taken from 10 averages (where these reported times include the acquisition of T_1_ and T_2_ maps). These datasets are referred to below as the “DW-SSFP-6” and “DW-SSFP-12” scans.

#### Structural protocol

The convergence of T_1_ values in fixed, *post-mortem* brain causes conventional T_1_ structural scans to have negligible gray-white contrast (Pfefferbaum et al., 2004). We instead use a 3D balanced SSFP pulse sequence (Miller et al., 2011) using TE/TR = 3.72/7.44 ms and *α* = 35°. This protocol results in high-contrast scans with inverted contrast compared to *in-vivo* T_1_-weighted structural scans (higher signal in gray matter than white matter). Balanced SSFP images are acquired in pairs with the RF phase incrementing 0° and 180°, which are averaged to reduce banding artifacts. Other parameters: 395 Hz/pixel, matrix size 576 × 576 × 480, 5/8 in-plane partial Fourier factor, 6/8 slice partial Fourier factor, voxel size 0.33 × 0.33 × 0.33 mm^3^ and acquisition time per pair of 32 min.

### Data analysis

All data were processed using the FSL software package (Smith et al., 2004) (specific tools are indicated parenthetically below). Individual acquisitions are co-registered using affine registration (FLIRT, (Jenkinson and Smith, 2001)) to correct for B_0_ drift and eddy-current distortions before averaging as described in (Miller et al., 2011).

#### Signal-to-noise calculations

We compared the predicted relative SNR efficiency for our DW-SE and DW-SSFP protocols to the SNR efficiency measured from the acquired data. For these measurements, diffusion-weighted contrast is effectively a confound, and it is easiest to compare the “unweighted” (low-b) scans (b = 0 s/mm^2^ for DW-SE and b_eff_ = 18 s/mm^2^ for DW-SSFP). Using our experimental parameters for the low-b DW-SE and DW-SSFP protocols and our default fixed tissue properties (T_1_/T_2_ = 400/45 ms, D = 0.08 × 10^− 3^ mm^2^/s), we predict an SNR efficiency ratio of *η*_SSFP_:*η*_SE_ = 2.77. (It is important to note that this high SNR efficiency gain reflects the use of shorter T_R_ at low b-value in DW-SSFP; the SNR efficiency gain at high-b is predicted to be a factor of 1.70, as described above. Thus, the value of 2.77 can be readily compared to experimental low-b data, but the relevant gain for tractography results is 1.70.) We measure the SNR efficiency in the low-b data in a whole-brain, white-matter mask generated by automated gray-white segmentation (FAST (Zhang et al., 2001)). Background noise measurements can be problematic in multi-channel data due to the additive effects of rectified noise (Dietrich et al., 2007). Instead, we use the repeated measurements at low-b (using all scans from our 24-hour protocols: n = 33 and 116 for DW-SE and DW-SSFP, respectively) to calculate the signal variance within each voxel. We average the SNR across the ROI and convert this into SNR efficiency by dividing by the square root of the volume scan time (0:50 for DW-SSFP and 6:22 for DW-SE).

#### Probabilistic tractography

The first stage of the probabilistic tractography uses Markov-chain Monte Carlo fitting to estimate the probability distribution for the fiber orientation in each voxel (BEDPOST, with a single fiber per voxel (Behrens et al., 2003)). For DW-SSFP, we use a modified version of this software described previously (McNab et al., 2009), which incorporates the DW-SSFP signal equation and T_1_ and T_2_ values to account for important differences in diffusion contrast between the two sequences (McNab and Miller, 2008). A second measure of data quality that can be calculated from the output of this stage of fitting is the 95% uncertainty on the orientation angle (*i.e.*, the 95% confidence angle from the mean direction) (Behrens et al., 2003). Tractography is performed in a second stage using a conventional probabilistic approach (Probtrack, (Behrens et al., 2003)). For every voxel in the seed mask, a series of streamlines are generated that, taken together, form a representation of the likely tract structure that incorporates the underlying uncertainty in the fiber orientation. Tractography used a step length of 0.2 mm, 5000 streamlines and no curvature threshold. Although tractography was performed in all four datasets, the most revealing results were based on comparison of DW-SE-6, DW-SE-12 and DW-SSFP-6. We therefore focus the discussion on these results, but provide the DW-SSFP-12 tract renderings in the supplementary material.

#### Tractography seed masks

Tractography was performed in the structural space using seed masks drawn on the structural image (shown in Fig. 12 of the supplementary material). For ease of visualization, the structural image was rigidly re-oriented so that the AC-PC line was present in an axial plane, and interpolated to 500 μm resolution. Four fiber tracks were chosen to represent all major tract types: projection (corticospinal tract, CST), commissural (corpus callosum, CC) and association (cingulum bundle and superior longitudinal fasciculus, SLF) tracts. The seed masks for each pathway was drawn on a single slice (described below), with no target or exclusion masks. Two seed masks for the CST (one in each hemisphere) were drawn in the posterior limb of the internal capsule in an axial plane approximately 5 mm superior to the AC-PC line at the coronal level of the most posterior part of the globus pallidus. Seed masks for the CC were drawn in the mid-sagittal plane, dividing the CC into 5 sub-regions based on the length fractions described in Hofer and Frahm (2006) (a revised definition of the Witelson (1989) sub-divisions). This definition aims to encapsulate the microstructural patterns of fiber density and diameter, and differentiate distinct cortical projection targets. Seed masks for the cingulum bundle (one for each hemisphere) were drawn in a coronal plane about 4–5 mm anterior to the posterior commissure. Seed masks for the SLF were drawn in the same plane, with the most medial part of the masks corresponding to the lateral aspect of the putamen. The only additional criterion for tracking was the use of a mask in the mid-sagittal plane to avoid cross-hemispheric tracking for the reconstruction of the left and right cingulum bundle (although we obtained very similar results without this additional mask when looking at combined left and right hemisphere results).

## Results

### Data quality comparison

Our first metric of raw data quality is the measured SNR efficiency, which can be compared to our predicted improvement by a factor of 2.77 in the low-b DW-SSFP data compared to the b = 0 DW-SE data. Measured SNR values in our whole-brain white-matter mask were 18.1 ± 5.6 and 18.6 ± 4.9. After accounting for the difference in scan times for DW-SE and DW-SSFP data, this yields in an SNR efficiency improvement for DW-SSFP over DW-SE of 2.94 ± 0.74. Given that the sequences are based on a common implementation of EPI but otherwise differ substantially, the mean value represents excellent agreement with theory.

**Fig. 4:**
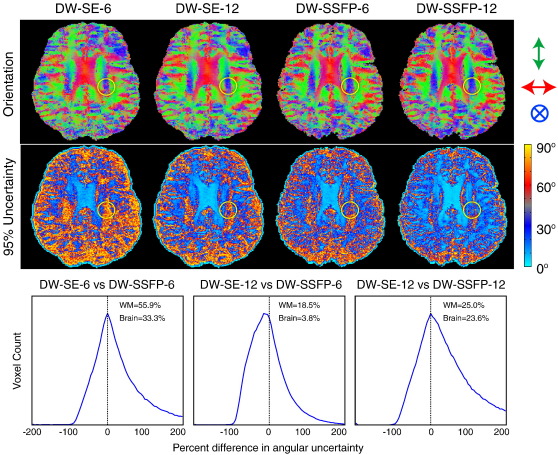
Comparison of color-coded fiber orientation vectors and the 95% uncertainty on the fiber angle using BEDPOST. Maps are axial slices, with the circles indicating crossing fibers in the centrum semiovale (relevant to the CST tractography results). The bottom plots show histograms of the voxel-wise percent difference in uncertainty for pairings of DW-SE and DW-SSFP data. Positive percent differences indicate lower uncertainty (better performance) for DW-SSFP (likewise, negative values reflect lower uncertainty for DW-SE). The inset text in each plot indicates the integral of the histogram (mean difference) within whole-brain (‘Brain’) and white matter segmentation (‘WM’) ROIs.

A second metric of data quality that is more specific to tractography is the 95% uncertainty in the orientation angle, which is shown in Fig. 4 along with the mean orientation map. All orientation maps are in broad agreement, although the DW-SE-6 data has noticeably higher noise. The difference between protocols is more clear with respect to the 95% uncertainty angle, where low uncertainty reflects high contrast-to-noise ratio. The DW-SE-6 has consistently highest uncertainty and DW-SSFP-12 the lowest, with fairly similar uncertainty for the DW-SE-12 and DW-SSFP-6.

These differences are quantified for three pairings of DW-SSFP and DW-SE scans in the histograms shown at the bottom of Fig. 4. The quantity being compared is the voxel-wise difference in angular uncertainty (normalized to give a percent difference), with positive values indicating lower uncertainty in the relevant DW-SSFP scan (and negative values *vice versa*). The histograms are drawn from the white-matter segmentation mask, and the inset text gives the average difference in uncertainty (*i.e.*, the integral under the histograms) for this mask and a whole-brain mask. For matched scan times (left and right histograms), DW-SSFP has overall much lower uncertainty. This is reflected in the high positive tails in the histograms, corresponding to 23.6–55.9% lower average uncertainty in DW-SSFP. The comparison of DW-SSFP-6 with DW-SE-12 is less clear from the histogram itself; the mean values calculated for the two masks indicate lower uncertainty in DW-SSFP in white matter (18.5%) but similar uncertainty in a whole-brain mask (3.8%).

### Tractography comparison

As mentioned above, we will limit our discussion of all tractography results here to the DW-SE-6, DW-SE-12 and DW-SSFP-6 datasets, since this best highlights the improvements afforded by DW-SSFP. For completeness, however, we include figures with matched results for DW-SSFP-12 in the supplementary material.

#### Corticospinal tract (CST, projection fibers)

**Fig. 5:**
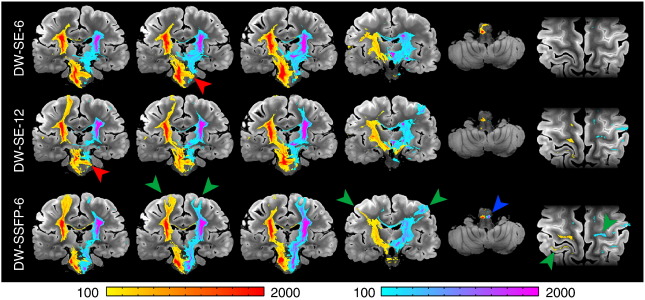
Comparison of tractography results obtained from right and left CST (seeded from the internal capsule) for DW-SE-6 (top), DW-SE-12 (middle) and DW-SSFP-6 (bottom) scans rendered with identical thresholding (≥ 100 streamlines). Generally, DW-SSFP-6 out-performs both DW-SE datasets. Some of the most significant differences can be seen in the dorsal tracts, with DW-SSFP-6 extending bilaterally into the sensorimotor cortices (green arrows) and the most caudal part of the medulla (blue arrow). In general, streamlines in the DW-SE data die out in the centrum semiovale where the CST crosses the CC and SLF. A small fraction of streamlines reach the sensorimotor cortex bilaterally for DW-SE-12, and unilaterally for DW-SE-6. There are also more false positives in the DW-SE datasets, for example, in the decussation of the superior cerebellar peduncles and the transverse pontine nucleus tracts (red arrows).

Virtual reconstructions of the CST from all three datasets are shown in Fig. 5. The reconstruction from DW-SSFP-6 was superior to DW-SE-6, and similar to (and in some areas better than) the DW-SE-12 tract reconstruction. Ventrally, the reconstructed CST descended all the way to the caudal medulla on both sides for DW-SSFP-6, but only on the right for DW-SE-6 and-12; the left CST died out in the pons for both DW-SE datasets. Prominent differences were also observed in the dorsal CST (green arrows in Fig. 5). The DW-SE-6 and-12 reconstructions were massively attenuated in the region of the centrum semiovale, where the CST crosses the CC and SLF (approximately 5 mm above the most dorsal part of the lateral ventricles). The CST was completely extinguished in this crossing fiber region in the DW-SE-6 data set, while a small fraction of streamlines from DW-SE-12 reach the primary sensorimotor cortex, premotor and supplementary motor areas. Conversely, in DW-SSFP-6, a large fraction of streamlines reached the same regions as DW-SE-12 bilaterally. We also observed several false positives in the CST for the DW-SE datasets (largely absent in DW-SSFP), including CST fibers crossing at the level of the decussation of the superior cerebellar peduncles and of the transverse pontine nucleus tracts. All datasets had some false positives that tracked into the cerebellum. When the results are rendered with no threshold on the number of streamlines, all datasets show a fraction of the streamlines entering the SLF or CC in the centrum semiovale. For DW-SE datasets, these false positives represent a similar number of streamlines as the true positives that follow the CST, while in DW-SSFP, the vast majority of streamlines correctly follow the CST in both hemispheres.

#### Corpus callosum (CC, commissural fibers)

**Fig. 6:**
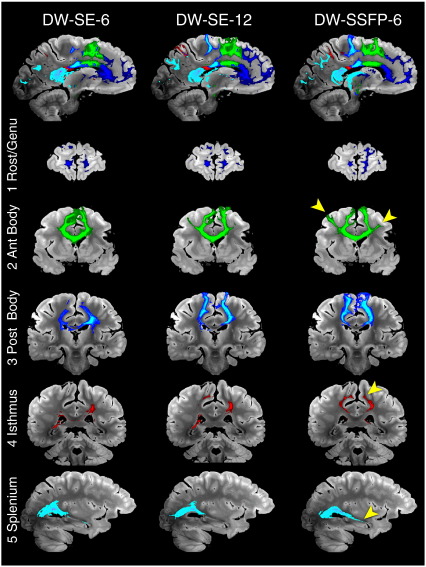
Comparison of tractography results obtained from five seed regions in the corpus callosum for DW-SE-6 (left), DW-SE-12 (middle) and DW-SSFP-6 (right) rendered with identical thresholding (≥ 1000 streamlines). The bottom five rows each depict a slice from consecutively numbered CC regions. Arrows indicate noteworthy features of the DW-SSFP-6 rendering, including: premotor cortex for the connections of the anterior CC body, somatosensory cortex in the connections of the isthmus and medial temporal lobe connections of the splenium.

Fiber tractography for the corpus callosum from our five masks is shown in Fig. 6. All three datasets overall reconstruct the divisions correctly, from rostral to caudal: (1) tracking from the rostrum/genu mask into pre-frontal cortex, (2) anterior CC body into pre-motor and supplementary motor cortex, (3) posterior CC body into primary motor cortex, (4) isthmus into parietal lobe and (5) splenium into occipital and temporal lobes. In general, DW-SSFP-6 tractography produced the highest-quality tract reconstructions, with tracts quite similar to the ones produced with DW-SE-12, and the poorest reconstructions obtained from the DW-SE-6 data. For the first sub-division (rostrum and genu), CC fibers tracked into pre-frontal areas BA9, 10, 11, 12 and the pars orbitalis with roughly equivalent success from all three datasets. In the second subdivision (anterior part of the CC body), DW-SSFP-6 out-performed DW-SE-12 in the number of streamlines reaching the supplementary motor and pre-motor cortices, while the DW-SE-6 dataset only tracked into the supplementary motor area. Streamlines starting from the third sub-division (posterior part of the CC body) reached the medial part of the primary motor cortex for all three datasets, although they penetrated most dorsally in the gray matter for DW-SSFP-6 followed by DW-SE-12, with no fibers reaching the lateral primary motor cortex for DW-SE-6. In the fourth subdivision (isthmus of the CC), streamlines reached the parietal cortex for all datasets, but only the DW-SSFP-6 dataset tracked into the left primary somatosensory cortex and the right precuneus. Finally, tractography seeded in the fifth subdivision (splenium) tracked into the occipital lobe and the precuneus, but it extended further in the medial temporal lobes in the DW-SSFP-6 dataset.

#### Superior longitudinal fasciculus (SLF, association fibers)

**Fig. 7:**
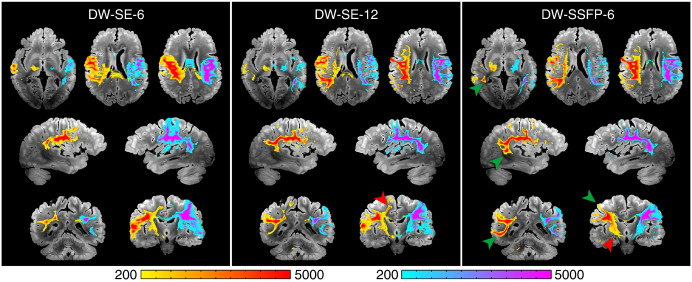
Comparison of tractography results obtained from right and left seed regions in the SLF for DW-SE-6 (left), DW-SE-12 (middle) and DW-SSFP-6 (right) rendered with identical thresholding (≥ 200 streamlines). Green arrows indicate areas of superior tract-tracing in the DW-SSFP dataset. Red arrows indicate false positives, including the ventral CST (arrow on the DW-SSFP-6 data, but found on all three renderings) and dorsal CST (prominent in the DW-SE data).

One clear example of DW-SSFP-6 surpassing DW-SE can be seen in the reconstructions of the SLF (Fig. 7). In both DW-SE datasets, the streamlines tracked only to the pars opercularis, while in the DW-SSFP-6 reconstructions, streamlines also extended bilaterally into the pars triangularis and pars orbitalis. More posteriorly, only the DW-SSFP-6 and DW-SE-12 datasets tracked into the supramarginal gyrus, the angular gyrus and the left precuneus. The tracts from DW-SSFP-6 going into the middle temporal gyrus were also more abundant than for DW-SE-12. More generally, we also observed far more false positives with both DW-SE datasets, especially extending to the corticospinal tract and corpus callosum.

#### Cingulum bundle (association fibers)

**Fig. 8:**
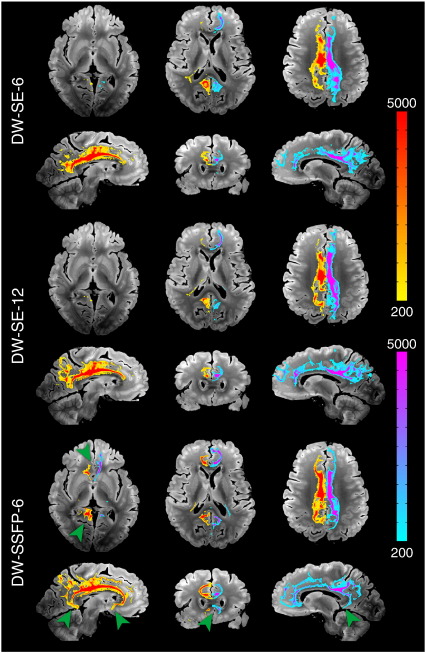
Comparison of tractography results obtained from right an left seed regions in the cingulum bundle for DW-SE-6 (left), DW-SE-12 (middle) and DW-SSFP-6 (right) rendered with identical thresholding (≥ 200 streamlines). All three datasets track the dorsal aspects of the cingulum with reasonable success. Prominent differences where DW-SSFP-6 is clearly out-performing both DW-SE datasets can be seen in the most anterior parts of the cingulum bundle and its temporal parts (green arrows).

**Fig. 9:**
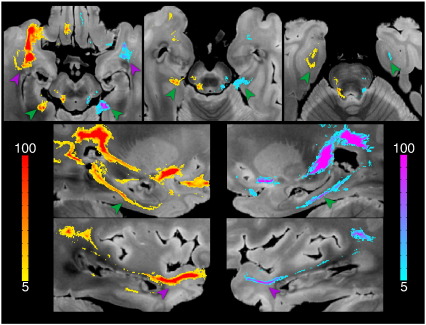
Zoomed regions depicting tractography of the cingulum bundle for DW-SSFP-6 into temporal regions, thresholded at 5–10 streamlines. The purple arrows indicate bilateral tracking into the parahippocampal gyrus, while green arrows indicate the connections to the subiculum of the hippocampus.

A striking example of the differences in data quality can be seen in the cingulum bundle (Fig. 8). The DW-SSFP-6 data produces a remarkably comprehensive representation of this tract compared to either DW-SE dataset. Only streamlines in DW-SSFP-6 reached bilaterally the septal nuclei and the basal forebrain. Cortical fibers of the medial superior frontal gyrus running in the cingulum bundle were tracked in the DW-SSFP-6 dataset, and in the DW-SE-12 data to a lesser extent. In the precuneus, cingulum fibers in the DW-SE-6 dataset died out in the posterior cingulate gyrus, whereas they extended to the medial BA1, 2, 3, 5 and 7 in DW-SSFP-6 and DW-SE-12. Finally, in DW-SSFP-6, streamlines could be reconstructed, remarkably, as far as the subiculum of the hippocampus and the parahippocampal gyrus bilaterally (see zoomed window in Fig. 9); none of these structures were reached by streamlines in any DW-SE data.

## Discussion

### Practical considerations for DW-SSFP

While our results are highly encouraging, several practical issues for use of DW-SSFP should be highlighted. The DW-SE sequence we use here, with a 3D segmented EPI acquisition, is more closely related to the conventional sequence used for *in-vivo* imaging. It may therefore be implemented with relatively minor changes to vendor-supplied diffusion sequences. DW-SSFP may in practice require slightly more effort to implement, but it is fundamentally a simpler sequence than DW-SE, involving fewer RF and gradient pulses (Fig. 3).

An important challenge for DW-SSFP is its intensive use of gradients, which leads to significant heating. As mentioned in the section Optimizing DW-SSFP, we found it necessary scale back our ideal acquisition parameters in order for the sequence to run reliably without over-heating (which, in our system, is detected and causes the scanner to stop). The use of a single-line readout with very low bandwidth would partly reduce heating without major loss of readout efficiency. However, the primary source of heating is the diffusion gradient: a 17 ms pulse at maximum amplitude that is repeated every 34 ms. By comparison, in the DW-SE sequence, the diffusion module takes about 100 ms and is applied every 530 ms, inducing negligible heating.

Finally, DW-SSFP has a different (and more complicated) signal dependence on the diffusion coefficient than DW-SE. Simply using the DW-SE equations with DW-SSFP data is likely to yield roughly the right orientation, but any estimates of uncertainty will be inaccurate (McNab et al., 2009). Hence, reasonable tract renderings have been demonstrated using deterministic tractography software without modification (Benner et al., 2009), but this approach is certainly not recommended for probabilistic tractography. A primary concern in this regard is the DW-SSFP signal dependence on T_1_ and T_2_: unlike DW-SE, this dependence cannot be removed through normalization with a b = 0 image. Given the variability in T_1_ and T_2_ reported for *post-mortem* brains, which appears at least in part to relate to tissue preparation, a conservative approach is to measure these properties and input the results to the fitting procedure. Our DW-SSFP imaging protocols therefore dedicated 32 min to mapping these relaxation times.

### Tractography results

Using the simplest procedure with a single seed mask drawn on a single slice, we were able to successfully reconstruct a major projection pathway (CST), the main commissural tract (CC) and two association pathways (cingulum bundle and SLF) in all diffusion datasets. For all tracts, DW-SSFP provided the best tractography results, both in terms of probability of connections and anatomically-meaningful spatial extent. Indeed, despite being acquired in half the scan time, DW-SSFP-6 provided tract reconstructions as good as, and often better than, DW-SE-12 data, while DW-SE-6 produced the poorest tractography.

Prominent examples where DW-SSFP-6 provided superior tract representation over both DW-SE-6 and-12 include: (i) number of CST fibers connecting the primary sensorimotor, premotor and supplementary motor cortex and CST fibers down to the caudal medulla (Nolte, 2009); (ii) fibers of the CC isthmus extending in the primary somatosensory cortex and precuneus bilaterally (Chao et al., 2009; Witelson, 1989); (iii) fibers of the cingulum bundle extending anteriorly to the septal nuclei and basal forebrain, and posteriorly to the parahippocampal gyrus and the subiculum bilaterally (Mufson and Pandya, 1984; Schmahmann et al., 2007), and (iv) number of SLF fibers connecting bilaterally with the pars triangularis and orbitalis (Schmahmann et al., 2007). Overall, DW-SSFP-6 therefore produced much fewer false negatives than DW-SE.

On the other hand, DW-SSFP-6 also produced far fewer false positives than DW-SE. For example, in the white matter tracts of the splenium, far less false positives can be seen in the fornix in DW-SSFP-6 compared to DW-SE-12. For the SLF, there were far less false positives tracking into the CST and CC in DW-SSFP. In the CST, we observed more false positives tracking into the anterior commissure in the DW-SE datasets, as well as some prominent false positives crossing via the decussation of the superior cerebellar peduncles and the transverse pontine nuclei tracts in both DW-SE datasets. The greater extent of the false positives, as with the true positives discussed above, is likely a consequence of the lower uncertainty in DW-SSFP. This kind of tractography error (false positive) is typically dealt with by the choice of appropriate exclusion masks, and represents more a fundamental challenge of diffusion tractography than a problem specific to our data.

We would like to re-emphasize that all the tractography procedures were carried out with no constraint on the curvature, using typical angular resolution (54 isotropically distributed gradient orientations were used) and, importantly, using a single seed mask drawn on a single slice. In the DW-SSFP dataset, despite the lack of masking constraint and standard angular resolution, we were able to reconstruct comprehensively pathways that prove difficult to track in *in-vivo* dataset such as the CST and the cingulum bundle, even when multiple steps using masking procedures are taken to track the entire pathway (Hagmann et al., 2003; Wakana et al., 2004, 2007). Given that the diffusion-weighted contrast is arguably lower in our data compared to *in-vivo* (since our b-value, although quite high, is not enough to compensate for the up to 10-fold reduction in diffusion coefficient), it seems likely that the quality of tractogtraphy is largely down to the high resolution of our data.

### Single-fiber tractography in crossing fiber regions

One consistent improvement in tractography with the DW-SSFP over DW-SE is the reconstruction of narrowing and crossing pathways, even with the use of a single-fiber BEDPOSTX fit. In the next section, we explore the ability of the data to support a second fiber population, demonstrating that the primary data sets reported here either do not support (DW-SE-6) or are on the cusp of supporting (DW-SE-12 and DW-SSFP-6) a second fiber. We therefore use single-fiber fitting for tractography because results are much more interpretable in this situation (compared to the situation when one data set fits a second fiber and another does not, or when the support for a second fiber is highly variable from voxel to voxel).

The CST fibers tend to die out in the DW-SE datasets where they cross with the CC and SLF in the centrum semiovale, particularly in the DW-SE-6 dataset. Similarly, streamlines originating from the seed masks in the body of the CC almost entirely died out in DW-SE datasets, particularly in DW-SE-6, where they meet with projection fibers, whereas they reached the lateral frontal lobe in DW-SSFP. Remarkably, the extremely thin anterior and ventral portions of the cingulum bundle (respectively reaching the septal nuclei and left basal forebrain on one hand, retrosplenial cortex as well as the left hippocampus and parahippocampus gyrus on the other hand) could only be reconstructed in DW-SSFP.

The observed differences in tractography are largely consistent with the observed uncertainty on fiber orientation, as shown in Fig. 4. The uncertainty in the estimate of the orientation is clearly lower in DW-SSFP-6 compared to DW-SE-6, and is estimated at 4–18% lower in DW-SSFP-6 compared to DW-SE-12. This causes the DW-SE streamlines to disperse over much shorter distances than DW-SSFP, which precisely benefit the reconstruction of narrowing tracts or pathways crossing other fiber bundles. Differences in quality of tractography in the CST is particularly revealing: inspection of the dispersion of the fiber orientation where the CST crosses the CC and SLF shows decreased uncertainty along the dorso-ventral path of the CST in DW-SSFP (circle in Fig. 4). The clear improvement in the DW-SE-12 data over the DW-SE-6 indicates that the difference between DW-SE and DW-SSFP is unlikely to reflect a differential sensitivity to different fiber populations, but rather suggests that the fitting procedure is simply less stable in these white matter areas due to lower SNR.

### Multi-fiber fitting in crossing fiber regions

**Fig. 10:**
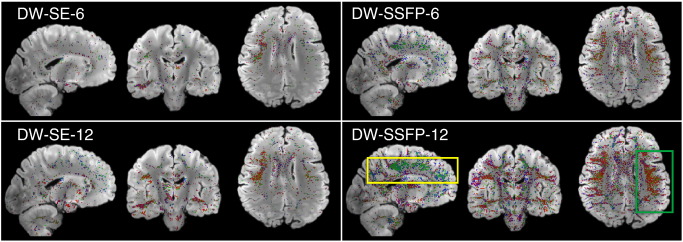
Maps indicating regions supporting estimation of a second fiber in DW-SE and DW-SSFP data sets. The volume fraction of the second fiber is shown using the standard direction color code, overlaid on the structural scan (*i.e.*, red is right-left, blue is superior–inferior, green is anterior–posterior). The overlay is thresholded to display regions where the data support estimation of a second fiber (according to automated relevance determination) and with fiber volume fraction was ≥ 0.05. As with tractography results, there is a major difference between DW-SE and DW-SSFP. Both DW-SSFP datasets support a second fiber across a range of known regions with multiple crossing fiber populations. The most prominent and spatially-extended examples are found where the SLF II crosses the CC (green box) and where SLF I crosses the superior corona radiata (yellow box).

Tractography in regions with multiple fiber populations can often be improved by fitting multiple fibers in each voxel (Behrens et al., 2007). The ability to estimate multiple fibers from *post-mortem* data has been demonstrated previously in animal brains (Dyrby et al., 2007, 2010) using high-performance, small-bore scanners. Further, although previous work has simulated the DW-SSFP signal in the presence of two fibers, the detection of multiple fibers has not been demonstrated. We investigated this possibility in all four datasets with a second BEDPOSTX analysis that includes a second fiber population, which is included only if its presence is supported by the data (using automated relevance determination). The regions supporting a second fiber population in our four datasets are indicated in Fig. 10. As with tractography results, a major difference between DW-SE and DW-SSFP data is observed. DW-SSFP-6 and-12 data support a second fiber with volume fraction *f*_2_ ≥ 0.05 in 9.0 and 16.8% of brain voxels, respectively, compared to 3.2 and7.0% in DW-SE-6 and-12.

**Fig. 11:**
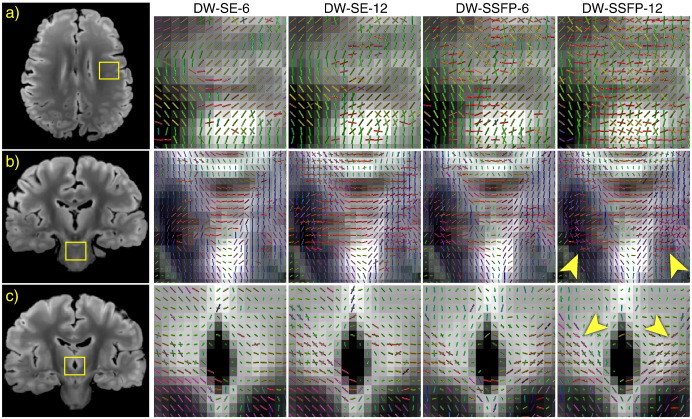
Two-fiber estimation in several regions with known decussational anatomy. In all cases, the second fiber population is most robustly estimated from the DW-SSFP-12 data. The DW-SSFP-6 and DW-SE-12 also support a second fiber in a reduced number of voxels, while the DW-SE-6 data set supports almost no second fibers. (a) An axial plane showing the crossing of the SLF II (green, running anterior–posterior) with the cortical projections of the CC (red, running right–left). (b) A coronal plane through the transverse pontine fibers (red, running right–left) in the presence of the CST (blue, running superior–inferior). (c) A coronal plane through the habenular commissure, a structure connecting the right and left habenular nuclei, is represented in the second fiber population (red–blue) while the primary fiber (green) is consistent with the stria medularis.

DW-SSFP datasets supported a second fiber in a range of known regions with multiple crossing fiber populations. The most prominent and spatially-extended example (boxes in Fig. 10) is found where the SLF II crosses the CC and the SLF I crosses the superior corona radiata. Some clusters of secondary fibers were estimated in the DW-SE-12, while the small number of voxels supporting a second fiber in DW-SE-6 were fairly isolated. Zoomed depictions of the reconstructed fiber architecture in several known regions of crossing fibers are shown in Fig. 11. Here, the DW-SSFP-12 dataset is most successful in capturing the known fiber architecture, while DW-SE-12 and DW-SSFP-6 have fairly comparable performance, and DW-SE-6 fails to properly reconstruct essentially any second fibers.

None of the datasets were able to resolve the centrum semiovale crossings where three populations co-exist (circles in Fig. 4), even when BEDPOSTX was run with a three fiber model. Furthermore, the two-fiber model generally resulted in higher angular uncertainty for the primary fiber compared to the single-fiber model. Nevertheless, these results suggest that with improved contrast and/or reduced noise, multi-fiber modeling could improve analysis from *post-mortem* human data.

## Conclusions

Diffusion imaging of human *post-mortem* brains is a recent topic of interest due to the potential to provide important validation of tractography techniques. However, the changes to *post-mortem* tissue due to death and fixation present significant challenges for data acquisition, particularly in human brains with long post-mortem intervals and immersion fixation. We have presented both theoretical predictions and tractography results demonstrating that DW-SSFP can perform significantly better under these altered tissue conditions, providing a powerful alternative to DW-SE for *post-mortem* diffusion tractography.

The following are the supplementary materials related to this article.

**Fig. 12:**
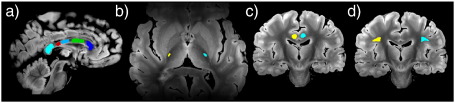
Supplementary material: Seed masks for tractography comparisons. Seed masks are drawn and displayed on the high-resolution structural image. Color-coded seed masks are shown for (a) corpus callosum, (b) posterior limb of the internal capsule (corticospinal tract), (c) cingulum bundle and (d) superior longitudinal fasciculus.

**Fig. 13:**
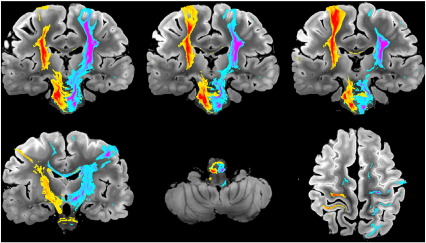
Supplementary material: CST tractography for the DW-SSFP-12 dataset.

**Fig. 14:**
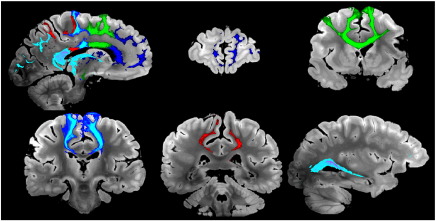
Supplementary material: CC tractography for the DW-SSFP-12 dataset.

**Fig. 15:**
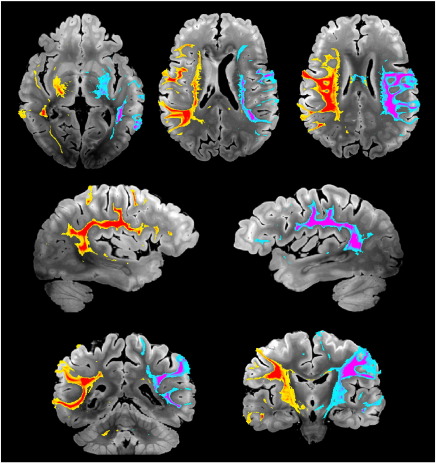
Supplementary material: SLF tractography for the DW-SSFP-12 dataset.

**Fig. 16:**
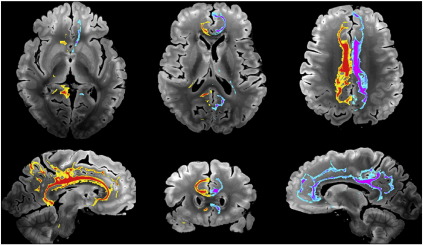
Supplementary material: cingulum bundle tractography for the DW-SSFP-12 dataset.

## Figures and Tables

**Table 1:** Optimal SNR efficiency for given b-values (for DW-SSFP, b_eff_). DW-SSFP is predicted to improve SNR efficiency over DW-SE by 48–127% (right-most column), depending on the desired b-value.

b (s/mm^2^)	Sequence	Signal (percent M_0_)	Readout efficiency (*ρ* × 100)	SNR efficiency (*η*)	*η*_SSFP_:*η*_SE_
1000	DW-SE	9.95	4.11	2.02	
DW-SSFP	4.63	41.74	2.99	1.48
3000	DW-SE	5.97	3.96	1.19	
DW-SSFP	3.64	36.33	2.19	1.84
5000	DW-SE	4.67	3.90	0.92	
DW-SSFP	3.14	32.35	1.79	1.94
10,000	DW-SE	2.80	3.77	0.54	
DW-SSFP	2.32	28.33	1.24	2.27

## Footnotes

1 The impact of T_acq_ on other aspects of image quality is an obvious concern. Here, we assume that the readout is multi-shot, such that we have flexibility in the fraction of k-space covered and T_acq_ does not constrain the total matrix size. For many multi-shot readouts like segmented EPI, T_acq_ describes the time taken to cover the extent of k-space. Thus, the phase accrual depends on T_acq_, but not on the density of k-space sampling per segment (*e.g.*, number of lines per segment). Thus, our optimization does not need to additionally specify bandwidth, echo spacing or equivalent measure to encapsulate off-resonance effects. The flexibility afforded by segmented readouts therefore enables us to optimize SNR in terms of T_acq_, taking image artifacts into account without having to specify in detail how k-space is covered.
